# Dental Section of the American Medical Association Meeting at Baltimore, Md., May 6th, 1895

**Published:** 1895-10

**Authors:** Vida A. Latham

**Affiliations:** Chicago


### THE DENTAL REGISTER.

VOL. XLIX.] OCTOBER, 1895. [No. 10.

### COMMUNICATIONS.

### Dental Section of the American Medical Association Meeting at Baltimore, Md., May 6th, 1895.

THE VALUE OF DIFFERENT DIAGNOSIS IN DENTISTRY BY DR. VIDA A. LATHAM, D.D.S., OF CHICAGO.

The subject of diagnosis is one of great importance and the man who can diagnosticate rapidly and correctly is usually successful in his profession. Diagnosis is valuable not only for treatment, but it enables one to form an accurate opinion as to the future course of a disease.

It seems a curious fact that works on dental surgery are so very imperfect and rambling on the subject of diagnosis. This is a matter of regret and also that research is so slow in progress—that is, slow in availing itself of so many new facts introduced from science generally. What is the reason? I am afraid it lies in five reasons:

1. The hurry to obtain a diploma.

2. The study of only just what seems absolutely necessary in the practice of dentistry and a corresponding inability to apply general principles.

3. The fear of encroaching on general medicine.

4. Insufficient preliminary education.

5. Lack of a thorough knowledge of the normal conditions, and a habit of relying too much upon one mode of treatment. The most difficult problems, perhaps, we have to diagnosticate in dental pathology are the neurological.

Dental irritation is one of the commonest and most powerful causes of reflex nervous disturbances.

We all know of many cases treated by the medical profession for months which might have been relieved by attention to the teeth. Understand, I do not mean to omit the medical treatment of such diseases as syphilis, malaria, neoplasmus, etc.

With regard to etiology of dental periostitis and pulpitis, the diplococus pneumonia may be regarded as a factor, usually accompanied by the staphylococus pyogenes aurens and albus.

Pulpitis may cause inflammation of the antrum just as well as a severe catarrhal inflammation can do so.

Causes of neuralgic pain are: 1, sensitive dentine; 2, fibroid pulps; 3, crowding; 4, necrosis of pulp in a confined space; 5, exostosis; 6, alveolar periostitis; 7, filling over exposed pulp; 8, malpresentation of third molar; 9, rhematic and gouty diathesis; 10, anemic and chlorotic states; 11, serumal calculus on roots; 12, malaria; 13, pulp nodules; 14, sympathy; 15, recession and absorption of gum and alveolus; 16, pressure of gases in the pulp chamber; 17, traumatism the most pernicious form of neuralgia is that which is due to septic influences occurring after some traumatic injury in which paresis or paralysis of the nerve affected sometimes follows. Differential diagnosis between antral abscess and ozœna —points in favor of the former are: 1, the presence of pulpless teeth; 2, a shortening of the face from the oral cavity to the orbit; 3, accumulation and reaccumulation of pus, showing at the hiatus, in middle meatus, half an inch from anterior extremity of inferior turbinated bone; 4, discharge of pus increased on putting patient in horizontal position especially on the sound side; 5, relative darkness over the diseased maxilla when the bones of the face are illuminated; 6, Puncture through nose and aspiration of fluid; 7, the presence of carious teeth, especially roots in the upper mandible.

Ozœna is recognized by: 1, characteristic fetid discharge; 2, olfactory anæsthesia; 3, detection of denuded necrosed bone in nares; 4, presence of crusts of dried secretions and ulcers, especially in naso-pharynx; 5, the teeth may be normal; 6, diathesis syphilitic or strumous.

The following appended tables may be useful in diagnosing:

| SENSITIVE DENTINE. | IF THE PULP IS SENSITIVE. |
|---|---|
| When an examination over a considerable part of the cavity walls does not respond to simple pressure, pain is not persistent. | When the examination is made near the pulp it responds to pressure. |

| HYPERÆMIA OF THE PULP. | PULPITIS. |
|---|---|
| Pain of boring character; tooth highly sensitive to hot and cold temperatures. Painful in mastication and on pressure. Hard to distinguish from. | Pain of a boring character rapidly increasing, assuming a throbbing form, extending from the diseased tooth to the neighboring teeth and to the side of the face, the tooth forming the center of its intensity. The larger and younger the pulp, the greater the pain. In time the pain subsides, to return, though, on the slightest provocation, or the horizontal position being resumed. Pulp injected with blood throughout, the exposed part deeper in color. |
| EXPOSED DENTINE. | |
| Absence of throbbing pain—pathogenomonic of pulpitis; serious neuralgia may be a symptom. | |

| CHRONIC PULPITIS. | NECROSIS OF PULP. |
|---|---|
| Pain less severe than in acute form, not very intense nor long in duration when present. Comes on at irregular intervals—often vague neuralgic pains. Sudden changes of temperature or applications of irritants will produce a paroxysm of pain lasting from a few minutes to hours. Pulp shows inflammation limited to exposed spots, but but the rest pale and healthy. | Pain becomes changed to a dull, heavy ache with a feeling of tension. Tooth feels too long, is raised in alveolus by the pericementitis and periostitis and loosened. May have some swelling at the side, on the gum or at the root. A change of color can be seen by strong light, the dead tooth having a dark line in the pulp region. |

It is often difficult to distinguish pulpitis from hyperæmia. In hyperæmia the throbbing character of the pain is not so well marked and the pulp usually not exposed. Pulpitis must also be distinguished from periostitis, the differential points being as follows:

| PULPITIS. | PERIODONTITIS. |
|---|---|
| Pain sharp, laminating or throbbing, intermittent and reflected within the tooth. | Pain dull, heavy and constant, and without the tooth. |
| Thermal changes cause pain. | Thermal changes do not cause pain. |
| Pressure or percussion on tooth gives no pain at first. | Pressure or percussion on tooth gives pain from the first. |
| Slight pressure on a piece of cotton in cavity generally gives acute pain. | Slight pressure does not give pain except through pressure transmitted to the periosteum. |
| With pulpitis we have pericementitis by continuity. | Tooth loosens and elongates. |

No diagnostician, who is conscientious as well as competent, will set up his own opinion as the standard but will always rely upon the perception of those to whom he wishes to make the diagnosis plain, and in this very idea is the safety; for the recapitulation of the case may reveal points previously unnoticed, so giving certainty and confidence to the patient and himself. To become a good diagnostician it is imperative to understand principles and laws, in preference to formularies and modes; and to comprehend these one's powers must be not only good but kept in constant use thus insuring the best and latest methods, reading and experience.

### THE SPECIFIC TREATMENT OF NECROSIS OF ALVEOLI AND MAXILLA WITH AROMATIC SULPHURIC ACID.

In his paper, Dr. W. A. Mills, of Baltimore, gave some of his experience with this old-time treatment. He cited a case of necrosis superinduced by chronic abscesses. The necrosis extended from the left central to the right bicuspid.

Treatment consisted of:

Acid sulphuric aromatic, - - ℥ij;

Aqua, - - - - - - - fl. ʒx;

To be injected into fistulous opening, by patient, five or six times per day. Bicarbonate of soda to be used as a mouth wash after each injection. At end of two weeks discharge ceased and the cavity was packed with:

Carbolic Acid, - - - - c.p.;

Tinct Iodin, - - - - aa ʒss;

Aqua, - - - - - - - fl. ℥xij;

The following day this dressing was removed, the nerve canals of devitalized teeth were opened, disinfected and filled. Patient instructed to syringe out cavity twice daily with the carbolic acid and iodin wash, as long as syringe could be used. In five weeks the parts were again in normal condition. The second case cited, the necrosis extended from the inferior cuspid to cuspid. Fistulous openings presented at the right of the left inferior cuspid and at the left of the right inferior cuspid and pus was copiously discharged. This condition had continued for over a year, although a number of physicians has prescribed for it. A surgeon told the patient that all of the incisor teeth would have to be removed before a cure could be effected. This the patient would not submit to. Treatment in this case was similar to Case No. 1 and cured. All the teeth were save intact.

In conclusion the essayist said: "The excuse I have to offer for my paper is to call the attention of the surgeon, no matter how prone he may be to operate in the oral cavity not to do so before he has failed with the sulphuric acid treatment, or use more conservative surgery than is usually practiced in similar cases."

### COMMON GROUND OF MEDICINE AND DENTISTRY.

Dr. Joseph Roach, of Baltimore, said:

While the work of the average dentist may be mechanical, the fact that both he and the surgeon work on the human body makes a bond of union between them which is worth study, inasmuch as whatever links in this bond are found to be the common property of both, are of much greater interest than any dissimilar points.

In the maladies of common interest, I wish to mention those often obscure and occasionally grave diseases that involve the maxillary sinus. This is obvious for the reason that in such cases the dental surgeon very often is apt to be the first observer on the ground. Whether he takes the cases himself or turns them over to the surgeon he should be able to accurately diagnose the trouble.

He spoke also of impacted and abscessed third molars often causing serious pharyngeal trouble.

He said: "The lower molar is often too large for the space between the second molar and the angle of the jaw. In consequence, its eruption is so interfered with that it may be either only partially erupted or may be impacted at an angle against the second molar appearing through the gum slightly or not at all. Abscess of this tooth often evolves not only the tissues immediately around it but the tissues of the throat becomes inflamed and grave results ensue. Abscess opening into the pharynx is certainly one of the sequellæ of this trouble."

### Chairman's Address.

Dr. M. H. Fletcher, of Cincinnati, in his address referred to the fact, that all the professions which have man himself for their subject, which aim to rectify his physical deficiences are today, and always have been, of greatest interest; their scientific advancement in the past decade is more marked than in any previous one, but our lack of knowledge is still very great.

In the course of his address the essayist said:

There is one feature of education, in both dental and medical colleges, which I consider of the greatest importance. It is that of having suitably trained instructors in our colleges. This I deem to be as great a defect as any at the present time, in our system of teaching.

It is not an uncommon thing to find in a class, students who outrank many of their professors in strength of intellect and natural ability. Not only these, but all students have a right to demand that their instruction be presented to them in the best possible manner.

It is a perfectly easy matter to get professors for the asking, but trained teachers are rare, and even those who have the natural qualities to become good instructors are not numerous. No one doubts that we have professors in almost every college who are thoroughly capable, but on the other hand a great number fall short of this standard. I believe a majority of students in all colleges are industrious and of good intent, and many of them are barely able, financially, to carry them through a three or four years' course.

Such students, if denied the full measure of their capabilities to receive, are defrauded of both money and time; the first of which never is, and the last never can be, repaid. There are no normal schools, so far as I know, for the proper training of dental and medical teachers, and if there were it does not seem practicable for many of our eminent practitioners—who are in other respects most suited for the position of instructors—to leave work and go to other localities for such training; but such a normal school, it seems to me, is demanded in some suitable form and it would seem a most fitting topic for discussion and action by our National

Association of Faculties. The distance a missle flies and the velocity with which it goes is exactly proportional to the force which impels it. The analogy holds good in our colleges, for students of high grade can not be graduated unless the force can be found in his Alma Mater to produce the desired result; consequently, it would seem no more necessary, if as much, to lengthen the term at college, increase the curriculum, and raise the standard of entrance, than it is to increase the quality and ability of college instructors.

### THE DESTRUCTION OF CHILDREN'S TEETH—CAUSE AND PREVENTION.

This paper was read by Dr. J. G. Henisler, of Baltimore. He attributed the destruction of teeth in children to neglect and ignorance as to the proper time and means of caring for the teeth. As a means of prevention he suggested that physicians watch the mouths of children and if decay appeared on the permanent teeth to send these patients to a dentist. Another means suggested was to educate patients themselves.

### ILL-DEVELOPED ORAL CAVITIES – IRREGULARITIES AND SOME OF THE CAUSES.

In his paper on this subject Dr. W. S. Twilley, Baltimore, said that among the causes of irregularity of teeth is the resistance offered the permanent teeth by the temporary ones. Another frequent cause is a want of simultaneous action between the increase of the permanent teeth and the decrease of the temporary, by absorption of their roots. When the permanent teeth are large and the growth of the jaws does not proceed proportionately, they are found to crowd and overlap. By premature extraction of the temporary teeth, the jaws are liable to contraction and when the permanent teeth appear, there not being sufficient room in the arches, they will crowd and overlap. Again, where the upper incisors extend inwards and come in contact with the lower centrals, the child finds it easier and more comfortable to throw the lower jaw forward.

This finally becomes habitual and promotes the increase in the length of the lower jaw itself. The almost universal acceptance of the theory, that the premature extraction of the deciduous teeth, or the extraction of the permanent ones, are the only causes of irregularities, is a great error. Many cases of irregularity are due to neoplasms and hypertrophies, as these growths in children cause a diminution of the normal contours of the bony framework of the oral cavity as well as that of the nasal.

### ARCH OR SPAN FILLING.

Dr. W. A. Mills, of Baltimore, read a paper on this subject. He described his method as follows:

Take, for example, two superior bicuspid teeth which have a space the sixteenth of an inch between them. I wish to fill with gold. In this case I have cavities on the proximal surfaces, otherwise I would make them. Prepare cavities as though they were to be filled separately. Be sure to make the anchorage as strong as possible as they become the abutments of the arch when completed. Set an orange-wood wedge firmly between the teeth. Bind teeth firmly together with german silver wire, to prevent any spreading of teeth during the packing of the filling material. Fill space with oxyphosphate mixed rather stiff, forcing sufficient cement over and against the buccal and palatine surfaces of the teeth to form a matrix. Before the cement sets too hard, remove it from cavities in the teeth, and a sufficient amount from the space to give proper form to the arch, buccal and lingual surfaces of the filling, then proceed to fill as though it were a single cavity. When filling is completed, remove rubber dam, matrix wedge, and binding wire then polish filling. With an amalgam filling the same process is used except that gutta-percha is employed in place of the oxyphosphate and this and the binding wire are not to be removed until twenty-four hours afterward when the filling can be dressed and polished.

I do not recommend these fillings only when necessity demands.

### CALCIFICATION OF THE TEETH, BY R. R. ANDREWS, CAMBRIDGE, MASS.

Calcification has always been a difficult subject, and authorities have been at sea concerning the finer process which nature takes to fashion the fully calcified substance.

Calcification is a process by which organic tissues become hardened by a deposition of salts of lime within their substance. In the intercellular tissue, and in the substance of the cells themselves, these salts are deposited by the rich blood supply always near. They are deposited in minute particles and in such fine subdivisions that it makes it difficult to demonstrate many of them even with the higher powers of the microscope.

The intercellular substance, either a protoplasmic or gelatinous fluid or semi fluid, contains the lime particles. In it they change their chemical nature, uniting with the organic substance of the part and form small globular bodies which have been called calco-spherites; and these blending or coalescing into a mass form a substance called calco-globulin. This calco-globulin, which is a lifeless matter, has been deposited through the cells into a gelatinous substance, and in some cases into the substance of the cells themselves, where, by a further hardening process, it becomes the fully calcified matrix. If a soluble salt of lime be slowly mixed with another solution capable of precipitating the lime, the resultant lime-salt will go down as an amorphous powder, and sometimes as minute crystals. But when the lime-salts are precipitated in gelatin or albumen, the character of the lime-salts is materially altered. Instead of a powder there were found various curious but definite forms, quite unlike the character of crystals or powder produced without the intervention of the organic substance. Mr. Rainey found that if carbonate of lime be slowly formed in a thick solution of albumen, the resultant salt has changed its character. It is now in the form of globules, laminated like tiny onions. These globules, when brought in contact with one another, become agglomerated into a single laminated mass, it appearing as if the lamina in immediate apposition had blended with one another. The globular masses at one time of mulberry-like form, lose the individualty of their constituent smaller globules and become smoothed down into a single mass, or longer, and Mr. Rainey suggests as an explanation of the laminated structure, that the smaller masses have accumulated in concentric layers, which have subsequently coalesced; and in the substitution of the globular for the amorphous or crystalline form in the salt of lime, when in contact with organic substances, Mr. Rainey claims to find the clue for the explanation for the development of shells, teeth and bone. At a more recent date Prof. Harting took up this line of investigation and found that other salts of lime would behave in a similar manner and that by modifying the condition of the experiment very various forms might be produced. The most important addition to our knowledge made by Prof. Harting lay in the very peculiar constitution of the "calco-spherites," by which name he designated the globular forms seen and described by Rainey. That these are built up of concentric laminæ, like an onion, has already been stated, and Mr. Rainey was aware that albumen actually entered into the composition of the globule, since it retained its form even after the application of acid. But Prof. Harting has shown that the albumen left behind, after treatment of a calco spherite with acid, is no longer ordinary albumen; it is profoundly modified, and it becomes exceedingly resistant to the action of acids, alkalies and boiling water. For this modified albumen, he proposes the name calco-globulin, as it appears that the lime is held in some sort of chemical combination, for the last traces of lime are retained very obstinately when calco-globulin is submitted to the action of acids. Now, it is a remarkable fact that microscopic glistening specks and globules are constantly seen at the edges of tissue where enamel cementum, dentine or bone are to be found or are forming. These microscopic globular bodies have been called calco-spherites, and it appears as though some such process, as described by Prof. Harting, is transpiring within the substance of the tissues where bone, dentine or enamel is to be formed. It will be noticed that near this point of formation there is always to be found a rich, capillary blood supply, and from this the lime-salts are given out. The abundant appearance of these microscopic glistening globules, referred to above, at the time of the formation of the enamel and their entire absence at earlier stages, is, to me, an indication that the globules are an enamel substance, the matrix forming calco-spherites, and following up their future confirms this.

The growth of the enamel once began takes place by addition of the globules. I am convinced that the larger ones are composed of hundreds of smaller ones which have coalesced into the main mass. When enamel is commencing its process of calcification, if we examine carefully with high powers, we shall find in that slight amount of the enamel organ that is directly over the calcified point of dentine, in what remains of the stellate reticulum and in the stratum intermedium principally a very large number of glistening points. They are the forming calco-spherites, or rather, they are the minute particles of lime from the blood supply, changing their chemical nature as they pass into the protoplasmic juices of the part. These appear to be passing into the formative cells and these cells superintend their formation into enamel rods—that is, they are laid from the cells against the forming rod. Within the substance of the ameloblasts they are seen to be growing larger by the smaller ones coalescing with others. If at this point of their development the layer of enamel cells is pulled away from the cap of the formed dentine, we shall see that the cap of dentine is everywhere covered with quite regularly formed granular bodies. If, on the other hand, the layer of enamel cells is against the formed cap of dentine the masses are assuming block-like forms, as though taking the form of the future enamel rod. They appear to be in a gelatinous substance which is between the dentine and the enamel cells, and here, by an unknown chemical process, they become the hardened columns of the enamel. In dentine, the calcifying process goes on in much the same manner.

The odontoblasts are merely masses of protoplasms and appear to have no membrane; as in the case with the ameloblast, it has a nucleus at a point farthest from the calcifying matrix. In forming the odontoblast, or pulp tissue through the odontoblast, it gives out a rich gelatinous substance about as wide as the layer of odontoblast cells. Everywhere between the odontoblasts is found a rich supply of connective tissue cells, whose function appears to be the forming of a network of connective tissue fibers into this gelatinous substance, this network seeming to be a scaffolding upon which the calco-spherites, which are to form calco-globulin, are to be deposited. Into this layer the odontoblasts are also superintending the placing of the minute globules, which are within them and which have been given to them the rich blood supply found everywhere near their pulp ends. Into the calcified substance the globules form against the calcified matrix where, fusing with others, they form a mass entirely filling the gelatinous substance. This gelatinous substance with its mass of globules now becomes calco-globulin. By some natural hardening process it then becomes calcified matrix and thus another layer of calcified matrix is formed.

In the cementum, a tissue I have not studied as carefully as I have the others, I am convinced that the calcifying process is much the same. The first cemental calcification takes place by the cementoblasts giving off these globular bodies near the neck of the rod into a gelatinous substance, this also being given off by the cells. It assumes the form of plates or scales, afterward the cells themselves appear to fill with the globules and lose their identity in the forming matrix. That peculiar tissue, which we call "tissue on the borderland of calcification," is composed of globular glistening bodies which have coalesced and formed a layer within a gelatinous substance previously given out by the formative cells. In this condition it is a tissue indestructible both in acids and in caustic alkalies and only in this condition is it true calco-globulin. The conclusions here given on the subject of calcification are arrived at after many years of original investigation. They are, I believe, with slight modification, accepted by most of the more recent authorities. I shall now review very briefly some of the, to me, erroneous views presented in a recent work—I refer to a work entitled, "The Anatomy and Pathology of the Teeth." The author and his associates, in a chapter describing "the calcification of the enamel," makes these statements:

"The more we turn to the center of the cup 'enamel organ' the more shall we be struck by the presence of glistering homogeneous lumps in the epithelia, until we have reached the center of the cup, where we observe that epithelium has been transformed into a number of such lumps in a regular arrangement, which reminds us of their origin, epithelia (enamel cells) gradually become enlarged and are at last split up into a number of medullary corpuscles." Again, "medullary tissue develops into connective tissue of a decidedly fibrous character." Again, "there is good reason for the assumption that the medullary tissue sprung from the previous external epithelium (of the enamel organ) is the source for the completion of such enamel as we observe upon temporary teeth when they emerge from their sockets." Again, "if we examine the lower edge of the cup of the enamel organ at about the sixteenth week of embryonal life we observe a peculiar change in the columnar bodies of the internal epithelium which consists in the appearance in a more or less row-like arrangement of highly glistening globular bodies replacing the previous columnar epithelia. These bodies are either solid or slightly vacuoled and *are formations of living matter* such as we are accustomed to look upon as medullary, embryonal or indifferent corpuscles, in their earliest stages of appearance. Obviously the glistening globules have originated from the reticulum of living matter of the columnar epithelia (enamel cells) themselves. We feel justified in this conclusion from the fact that we can trace, step by step, the growth of these glistening granules up to the formation of glistening lumps such as we have termed medullary corpuscles. * * * * The lumps are extremely glossy, with a high degree of refraction. They are arranged at first irregularly in a layer of considerable breadth, and higher up in rows, and by their coalescence and prolongation give rise to small columns, the ameloblasts. * * *

"These (medullary) corpuscles or the liquids contained in their reticulum become solidified into basin substance and immediately infiltrated with lime-salts. * * * The enamel rods are built up in rows of such calcified or petrified medullary corpuscles."

These observations, in regard to the calcification of the dentine, endeavor to show that the odontoblasts are split up at their distal ends into these glistening bodies, which they call medullary corpuscles, are lumps of protoplasm in which living matter is stored up in different shapes, the glistening globules of small size having arisen from protoplasm, and that these represent a juvenile condition of living matter in its most compact aggregation which enter directly into the formation of the basis substance of dentine, while at the same time, continually superadded to the proximal ends of the odontoblasts are the medullary corpuscles derived from the living matter of the papilla. The continuity of the odontoblasts in dentine is established. They assert a similar proceeding from ameloblasts in a reverse direction. Thus, the ameloblasts being broken up at their proximal (dentine) ends into medullary corpuscles, which are entirely transformed into blocks of enamel rods and superadded to at their distal or perispherical ends by medullary corpuscles dérived from the stratum intermedium. * *

"The indifferent corpuscles serving to supply additions to the ameloblasts exhibit all intermediate stages between small, globular, glossy and compact nucleated, protoplasmic lumps." * * "Nothing but a transmutation of solid, globular lumps of living matter in delicately reticulated medullary corpuscles seem to be required for the building up of the minute blocks of the enamel rods without the intermediate stage of ameloblasts. * * * The first appearing enamel is made up of irregular, angular, glistening lumps, greatly varying in size."

In these few selections from a chapter in this book on "Calcification" I have given some of the points which they present, and these I propose to briefly review. No one can be more clearly aware of the patient and persistent effort and of the immense amount of labor and earnest research which the author has given to his work than myself, and great credit from his profession is due to Dr. Bodecker for his labor. I am not in accord with his views as to calcification of the dental tissues. To some of us "the reticulum" and the "medullary corpuscles" are bugbears. To the earnest investigator, who did not know the author, it would seem from their description of the calcifying processes, as if a tissue has been built up to fix a theory. So far as I am aware, photo-micrographs of these tissues, as described in this chapter on calcification, have never been shown. There is no absolute evidence to prove the correctness of their assertions. These peculiar theories on the calcifying process cause a very considerable amount of doubt in the mind of any one who has given this subject attention in the way of original investigation. The theories advanced clash strangely with facts. The glistening bodies seen in the epithelial layers of the enamel organ are but lifeless lime globules and do not have their origin in a reticulum of living matter in these epithelial layers. Their origin is more probably from the blood supply which is everywhere abundant near these layers. It is speculating in a very lively manner to assert that the cells in the enamel layers split up into a number of medullary corpuscles of a fibrous character and then become formations of living matter; and it is wholly a hypothetical statement to make when they say that these glistening bodies by coalescing and prolongation give birth to the ameloblasts. These lumps of living matter which they call medullary corpuscles are but glistening masses of lifeless matter known to be calco-globulin.

They are not medullary corpuscles; they do not arise from protoplasm; they are not "a juvenile condition of living matter in its most compact aggregation."

